# Aggressiveness of Care at the End-of-Life in Cancer Patients and Its Association With Psychosocial Functioning in Bereaved Caregivers

**DOI:** 10.3389/fonc.2021.673147

**Published:** 2021-06-04

**Authors:** Justus Tönnies, Mechthild Hartmann, Dirk Jäger, Caroline Bleyel, Nikolaus Becker, Hans-Christoph Friederich, Markus W. Haun

**Affiliations:** ^1^ Department of General Internal Medicine and Psychosomatics, Heidelberg University, Heidelberg, Germany; ^2^ Department of Medical Oncology, National Center for Tumor Diseases, Heidelberg University Hospital, Heidelberg, Germany; ^3^ Department of Child and Adolescent Psychiatry, Heidelberg University, Heidelberg, Germany; ^4^ Division of Cancer Epidemiology, German Cancer Research Center, Heidelberg, Germany

**Keywords:** aggressiveness of care, regret, mental health, cancer, caregivers, multivariate analysis of variance

## Abstract

**Study Registration:**

https://www.drks.de/drks_web/navigate.do?navigationId=trial.HTML&TRIAL_ID=DRKS00022837,DRKS00022837.

**Background:**

Intensified oncological treatment for advanced cancer patients at the end-of-life has been specified as aggressiveness of care (AOC) and increased over the past decades. The aims of this study were to 1) determine the frequency of AOC in Central Europe, and 2) investigate differences in mental health outcomes in bereaved caregivers depending on whether the decedent had experienced AOC or not.

**Materials and methods:**

We conducted a cross-sectional study in a large tertiary comprehensive cancer care center in Germany. Bereaved caregivers provided information about (a) treatment within the last month of life of the deceased cancer patient and (b) their own mental health status, *i.e.*, decision regret, complicated grief, depression, and anxiety. After multiple imputation of missing data, differences in mental health outcomes between AOC-caregivers and non-AOC-caregivers were analyzed in a multivariate analysis of variances.

**Results:**

We enrolled 298 bereaved caregivers of deceased cancer patients. AOC occurred in 30.9% of all patients. In their last month of life, 20.0% of all patients started a new chemotherapy regimen, and 13.8% received ICU-treatment. We found differences in mental health outcomes between bereaved AOC- and non-AOC-caregivers. Bereaved AOC caregivers experienced significantly more decision regret compared to non-AOC caregivers (Cohen’s d = 0.49, 95% CI [0.23, 0.76]).

**Conclusion:**

AOC occurs frequently in European health care and is associated with poorer mental health outcomes in bereaved caregivers. Future cohort studies should substantiate these findings and explore specific trajectories related to AOC. Notwithstanding, shared-decision making at end-of-life should increasingly account for both patients’ and caregivers’ preferences.

## Introduction

While some patients benefit from innovative treatments for advanced cancer (1–3), an increasing number of patients are intensively and aggressively treated until the end of life (4–9). The concept aggressiveness of care (AOC) summarizes this development and is defined by the following empirically identified factors: 1) start of a new chemotherapy regimen within the last month of life, 2) chemotherapeutic treatment within the last 14 days before death, 3) more than one emergency department visit within the last month of life, 4) more than 14 days hospitalization within the last month of life, 5) more than one hospital admission within the last month of life, and 6) admission to intensive care unit (ICU) within the last month of life (10). Most of the findings on AOC occurrence originate in the Anglo-American and East Asian region. For European health care systems, which are more often publicly financed than the US system, data on AOC occurrence and trends of AOC frequencies are scarce. Although, initial studies point to a similar trend of increasing aggressive treatments in advanced-stage cancer patients (11–13), it is still unclear whether there are differences in AOC occurrences when patients have statutory health insurance instead of being privately insured (14). Further differences between the US and many other European health care systems regarding reimbursement of treatment costs, availability of variant treatment options, and the population structure may play an important role in the use of health care services in general, but at the end of life in particular (15). Contrary to the assumption that more intensive treatment necessarily leads to better outcomes, recent studies indicate that AOC is associated with a lower quality of life in patients suffering from advanced cancer. Specifically, AOC is linked to reduced survival times, more admissions to hospitals, increased receipt of life sustaining treatments, and more frequent dying in an intensive care unit (16–19). The reasons and motives why an increasingly large number of patients receive AOC are complex and often related to the subjective values of the patient, his or her caregivers, and the treating physicians. From the patients’ perspective, caregivers’ opinions play an important role in treatment decision-making that may or may not culminate in AOC (20). In this regard, caregivers tend to favor the prolongation of potentially curative treatment which is related to increased likelihood of AOC at end-of-life (21). The intensity of treatments which advanced-stage cancer patients receive is associated with the way bereaved caregivers perceive the quality of care at the end of life (20). In a large interview study bereaved caregivers of 1,146 patients reported less quality of care when AOC was observed (22). Furthermore, there is preliminary evidence that aggressive treatment in advanced cancer patients may be associated with a higher risk of psychological distress in bereaved caregivers (23, 24). However, there still is only little data on frequency of occurrence of AOC at end of life in Central European advanced cancer care, and especially its relation to psychological outcomes among (bereaved) caregivers is largely unknown.

Hence, this cross-sectional study aimed first to estimate the frequency of AOC as defined above in a large, tertiary comprehensive cancer care center in Germany. In a second step, we aimed to examine differences in mental health outcomes of bereaved caregivers, *i.e.*, levels of depressive symptoms and anxiety, complicated grief, and decision regret, depending on whether the decedent had experienced AOC or not.

We hypothesized that bereaved caregivers who had witnessed AOC showed a significantly higher mental health burden compared to caregivers who had not witnessed AOC.

## Materials and Methods

### Study Design and Setting

This cross-sectional study comprised a survey in bereaved caregivers of cancer patients who had received their treatment at the National Center for Tumor Diseases (NCT) in Heidelberg, a large, tertiary comprehensive cancer care center in Germany. The period of recruitment and data collection was between October 2015 and March 2016. The study was funded by the German Research Foundation (DFG) and approved by the Institutional Review Board of the Medical Faculty of Heidelberg University (Registration-No. S-500/2014). Prior to enrolment, all participants were able to ask questions and required to give their informed consent.

### Participants

For our sample we sought to contact the bereaved caregivers of cancer patients registered in the Cancer Registry of the NCT Heidelberg who had died between June 30^th^, 2012 and December 31^st^, 2014. The caregivers approached were listed as contact person by the patients. Inclusion criteria for caregivers were: 1) aged 18 years or older, 2) patient had passed away at least six months prior to caregiver’s study participation, and 3) patient was diagnosed with the following tumor entities: pancreatic, prostate, colon, breast, and lung cancer. Exclusion criteria were: 1) insufficient German language proficiency, 2) cognitive and/or physical impairment, which precluded adequate completion of the questionnaires, and 3) participation in an oncological trial which led to chemotherapy. In a first step, we reviewed the patient’s medical record for information about a contact person. Additionally, we checked the patient’s date of death and whether she or he was diagnosed with one of the included tumor entities. In a second step, we called the identified caregivers initially by phone and determined age, language proficiency, and capability of filling in questionnaires and asked whether the patient participated in an oncological trial in which chemotherapy had been prescribed. If caregivers refused study participation, we recorded the reason for non-participation. We mailed an invitation letter, the study information material, and the set of questionnaires comprising sociodemographic and medical data and psychometrically validated instruments to all interested caregivers and offered a telephone appointment to answer questions raised from the documents. After we clarified all the questions, caregivers filled in the set of questionnaires and mailed it back to the study center along with the signed consent form. For study participation, we chose the timepoint of at least 6 months after the patient’s death so that caregivers would be likely to be beyond a state of acute grief (25), but close enough to the death to minimize the risk of recall bias. For estimating the frequency of AOC in patients, caregivers provided sociodemographic and medical data including treatment modalities in the last weeks (*e.g.*, receiving chemotherapy, admissions to hospital and/or ICU and/or emergency departments). To investigate differences between AOC in patients and psychological outcomes in bereaved caregivers, caregivers completed psychometrically validated instruments. We reminded not responding caregivers up to four times *via* telephone and mail (twice each) and recorded reasons for non-participation.

To achieve a power of 80 at a Type I error rate of 5% when assessing small effects, we estimated that approximately 100 participants per group would be required in a two-group design applying four dependent mental health outcomes measures (26). To account for missing data in a potentially highly vulnerable population, we inflated the sample size to 300 participants.

### Variables

To adapt the definition of AOC to the German health care system where short and medium-long hospital admissions are more common than in the US, we shortened the list of determining factors. We considered the following aspects as the key factors for the definition of AOC usage: 1) new chemotherapy regimen starting less than 30 days before death, 2) the last dose of chemotherapy within 14 days of death, and 3) more than one day ICU stay within the last month of life. If at least one of these factors had been present, we defined AOC usage as present. The occurrence of AOC was considered as independent variable for all the analyses.

As dependent variables, we first assessed constructs assumed to be more strongly related to AOC. Specifically, we measured regret after treatment decisions by using the Decision Regret Scale for Caregivers (DRS-C) (27). The DRS-C is the caregiver version of the Decision Regret Scale originally developed for patients by Brehaut and colleagues (28). It is a unidimensional, self-report instrument consisting of five items, which are answered on a five-point bipolar intensity scale ranging from *strongly agree* to *strongly disagree*. The participants are asked to retrospectively evaluate treatment decisions that were made at the end of the patient’s life. To determine whether the caregivers experienced potentially maladaptive aspects of grief, we used the 19-item Inventory of Complicated Grief (ICG) (five-point Likert scale ranging from *never* to *always*; higher scores indicate higher levels of complicated grief) (29). Second, we assessed more general mental strains, namely depression and anxiety by using the Patient Health Questionnaire 9 (PHQ-9) (scores range from 0 to 27 with 5, 10, 15, and 20 indicating mild, moderate, moderately severe, and severe levels of depressive symptoms) and the Generalized Anxiety Disorder Scale (GAD-7) (scores range from 0 to 21, with 5, 10 and 15 representing mild, moderate, and severe levels of anxiety symptoms), respectively (30).

### Statistical Methods

For descriptive statistics, we used frequencies and proportions for categorical variables and means or medians for metric variables. To detect differences in mental health outcomes of bereaved caregivers, *i.e.*, levels of depressive symptoms, anxiety, complicated grief, and decision regret, depending on whether the decedent had experienced AOC or not, we conducted a one-way multivariate analysis of variance (MANOVA). Prior to the main analysis, we eliminated multivariate outliers using Mahalanobis distance to limit their effect on the Type I error. We then performed multiple imputation for participants who had <10% missing values (n = 35) on continuous variables (*m* = 8 imputed sets with 30 iterations) (31). We evaluated assumptions (independence, linearity/additivity, normality, and equality of variance-covariance matrices) prior to conducting our main analysis (see **Supplementary Material**). For the MANOVA, the robust Pillai’s criterion was chosen to increase statistical power and control the family-wise error rate of intercorrelated dependent variables. For *post hoc* comparisons, we conducted Games–Howell tests to account for potential heterogeneity in variance between groups. To control for specific caregivers’ and patients’ characteristics we further included gender of the bereaved, relationship to the deceased (spouse/partner *vs* other), the patient’s age at time of death, and the time between death and study participation as covariates (only main effects) in the MANOVA. The statistical analysis of the data was conducted using R, version 4.0.3 (32). For all analyses, statistical significance was evaluated at a Type I error of 5% (two-tailed).

## Results

### Participants

**Figure 1:**
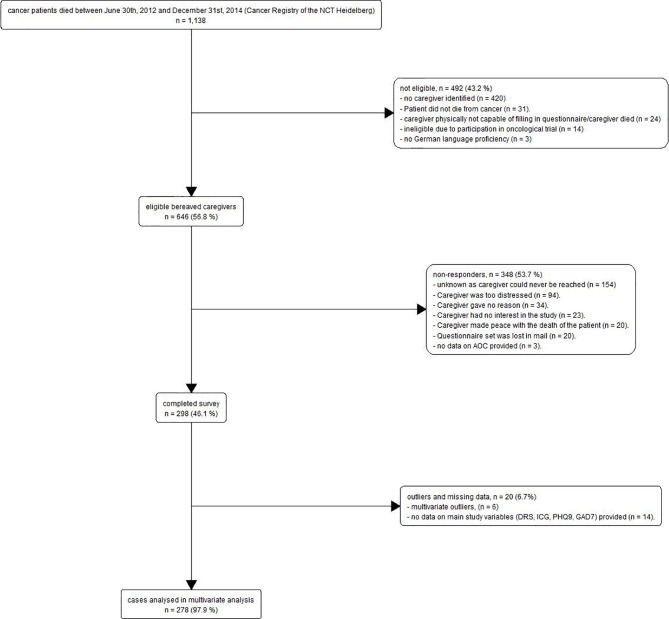
Study Flow Chart.

From 1,138 patients who were detected in the Cancer Registry of the NCT Heidelberg and died between June 30th, 2012 and December 31st, 2014, we were able to identify 646 potentially eligible bereaved caregivers. Among the eligible caregivers, 348 non-responders could not be included due to several reasons, mostly because the caregiver could never be reached (n = 154) or was too distressed to participate (n = 94). In total, 298 caregivers completed the questionnaires (response rate: 46.1%). After removal of six outlier cases and 14 participants with missing data on all main study variables (ICG, GAD7, PHQ9, DRS-C), we retained 278 observations for the multivariate analysis. Complete information on all the main study variables was available for 258 participants. For the study flow diagram see **Figure 1**. For the detailed sample characteristics of the 298 responders and the descriptive statistics as well as Pearson product-moment correlations for the study variables see **Tables 1** and **2**, respectively. There were no significant differences between participants and non-responders concerning gender, χ²(1, N = 452) = 0.00, p =.982), the only characteristic available from non-responders.

**Table 1:** Characteristics of participants.

	AOC^a^ absent (N = 206)	AOC^a^ present (N = 92)	Overall (N = 298)	Comparison^d^
**Age**				p=.146
Mean (SD)	62.3 (13.6)	59.43 (16.5)	61.4 (14.6)	
Median [Min, Max]	64.0 [30.0, 90.0]	62.0 [18.0, 90.0]	64.0 [18.0, 90.0]	
Missing	13 (6.2%)	1 (1.1%)	14 (4.7%)	
**Gender**				p=.782
Female	123 (59.7%)	54 (58.7%)	177 (59.4%)	
Male	78 (37.9%)	38 (41.3%)	116 (39.0%)	
Missing	5 (2.4%)	0 (0%)	5 (1.7%)	
**Relationship status (at study participation)**				p=.255
Single	126 (61.1%)	67 (72.8%)	193 (64.8%)	
In a relationship/married	60 (29.1%)	22 (23.9%)	82 (27.5%)	
Missing	20 (9.7%)	3 (3.2%)	23 (7.7%)	
**Employment status (at study participation)**				p=.326
Employed	61 (29.6%)	36 (39.1%)	97 (32.6%)	
Retired	109 (52.9%)	44 (47.8%)	153 (51.3%)	
Domestic work/student	6 (2.9%)	4 (4.3%)	10 (3.4%)	
Unemployed	6 (2.9%)	1 (1.1%)	7 (2.3%)	
Other	5 (2.4%)	2 (2.2%)	7 (2.3%)	
Parental or sick leave	5 (2.4%)	3 (3.3%)	8 (2.7%)	
Missing	14 (6.8%)	2 (2.2%)	16 (5.4%)	
**I am the … of the deceased**				p=.675
Partner	146 (70.9%)	62 (67.4%)	208 (69.8%)	
Parent	4 (1.9%)	3 (3.3%)	7 (2.3%)	
Sibling	3 (1.5%)	2 (2.2%)	5 (1.7%)	
Child	42 (20.4%)	23 (25.0%)	65 (21.8%)	
Grandchild	2 (1.0%)	1 (1.1%)	3 (1.0%)	
Other	4 (1.9%)	0 (0%)	4 (1.3%)	
Missing	5 (2.4%)	1 (1.1%)	6 (2.0%)	
**Age of deceased in years**				p =.047
Mean (SD)	68.0 (10.4)	65.4 (10.3)	67.2 (10.5)	
Median [Min, Max]	69.0 [38.0, 102]	65.5 [37.0, 88.0]	68.0 [37.0, 102]	
**Tumor diagnosis of the patient**				p =.037
Lung	84 (40.8%)	32 (34.8%)	116 (38.9%)	
Pancreas	51 (24.8%)	37 (40.2%)	88 (29.5%)	
Breast	28 (13.6%)	15 (16.3%)	43 (14.4%)	
Prostate	27 (13.2%)	4 (5.4%)	31 (10.4%)	
Colon	15 (7.3%)	4 (4.3%)	19 (6.4%)	
Trachea	1 (0.5)	0 (0%)	1 (0.4%)	
**Time between initial tumor diagnosis and death of deceased in months**				p =.102
Mean (SD)	30.1 (35.2)	21.9 (38.1)	27.6 (36.2)	
Median [Min, Max]	18.0 [0, 217]	11.0 [0, 284]	15.0 [0, 284]	
Missing	24 (11.7%)	12 (13.0%)	36 (12.1%)	
**Time between death of deceased and study participation in months**				p =.051
Mean (SD)	25.1 (7.56)	27.2 (8.81)	25.8 (8.01)	
Median [Min, Max]	25.0 [12.0, 46.0]	27.5 [11.0, 14]	26.0 [11.0, 46]	
Missing	–	–	–	
**Level of depressive symptoms (PHQ-9)** ^b^				p =.234
Blank	44 (21.4%)	19 (20.7%)	63 (21.1%)	
Mild	63 (30.6%)	23 (25.0%)	86 (29.9%)	
Moderate	39 (18.9%)	23 (25.0%)	62 (20.8%)	
Severe	29 (14.1%)	8 (8.7%)	37 (12.4%)	
Highly severe	12 (5.8%)	13 (14.1%)	25 (8.4%)	
Missing	19 (9.2%)	6 (6.5%)	25 (8.4%)	
**Level of generalized anxiety (GAD-7)** ^c^				p =.484
Blank	71 (34.5%)	29 (31.5%)	100 (33.6%)	
Mild	59 (28.6%)	26 (28.3%)	85 (28.5%)	
Moderate	33 (16.0%)	21 (22.8%)	54 (18.1%)	
Severe	24 (11.7%)	10 (10.9%)	34 (11.4%)	
Missing	19 (9.2%)	6 (6.5%)	25 (8.4%)	

^a^AOC, Aggressiveness of Care; ^b^PHQ-9, Patient Health Questionnaire 9; ^c^GAD-7, Generalized Anxiety Disorder 7; ^d^For comparisons on continuous variables, t-test for independent samples were conducted, for discrete variables, χ^2^-tests were conducted.

**Table 2:** Descriptive statistics and pearson product-moment correlations for study variables.

Variable	*N*	*M^a^*	*SD^b^*	*K^c^*	*Sk^d^*	1	2	3	4
1. Decision regret	274	36.6	22.93	2.81	.36*	–			
2. Complicated grief	270	46.2	14.14	2.44	.39*	.30*	–		
3. Level of depressive symptoms	273	9.49	6.11	2.27	.44*	.23*	.61*	–	
4. Level of anxiety	273	7.35	5.19	2.61	.67*	.19*	.48*	.79*	–

*p <.01; ^a^M, mean; ^b^SD, standard deviation; ^c^K, Kurtosis; ^d^Sk, Skewness.

### Frequency of AOC

**Figure 2:**
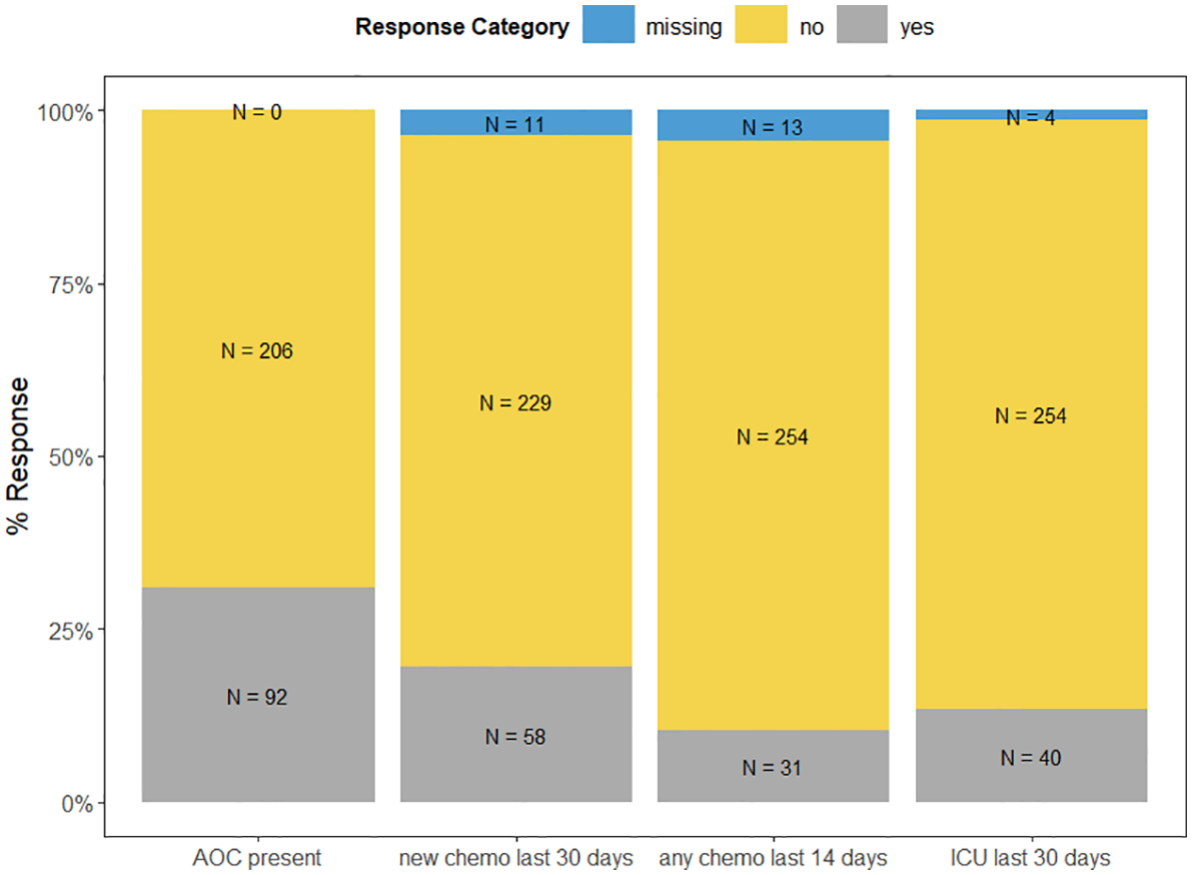
Frequencies of AOC Components.

Overall, 30.9% of the caregivers (92/298) reported that their deceased family member had experienced at least one component of AOC at the-end-of-life. Specifically, the initiation of a new chemotherapy regimen within the last month of the patient’s life was the most frequently experienced component of AOC (19.50% of all patients), followed by treatment on ICU (13.4% of all patients), and receipt of chemotherapy in the last 14 days of the patient’s life (10.4% of all patients). For a detailed display of frequencies of AOC components see **Figure 2**.

### Mental Health Outcomes of Bereaved Caregivers Depending on the Experience of AOC

**Figure 3:**
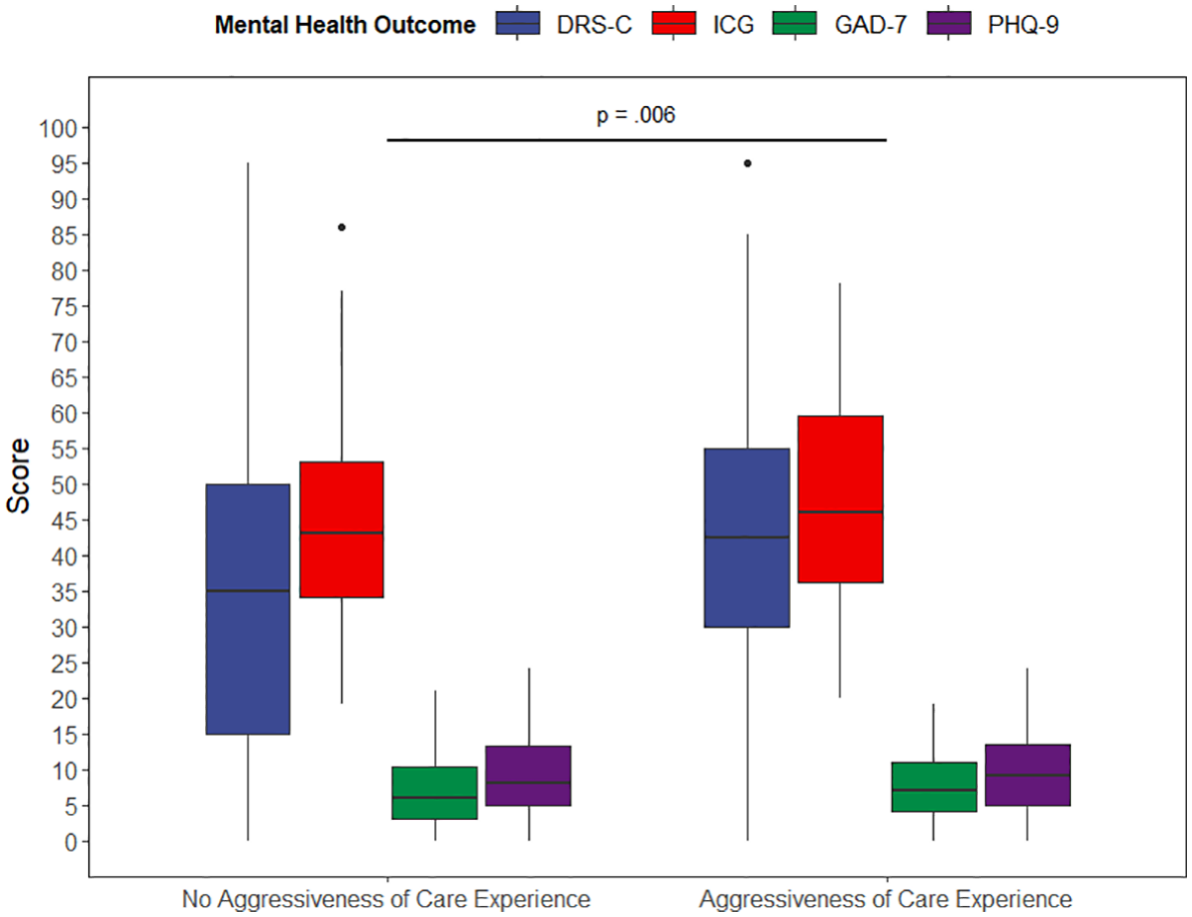
Results of the Multivariate Analysis of Variances (MANOVA). Note: *MANOVA, F (4*,253) *= 3.75, p = 0.006, partial eta-squared = 0.06, n = 278*; Games–Howell method for pairwise comparisons. Adjusted p-value using Tukey’s method. DRS-C, Decision Regret Scale for Caregivers; ICG, Inventory of Complicated Grief; GAD-7, Generalized Anxiety Disorder Scale -7; PHQ-9, Patient Health Questionnaire-9.

A one-way MANOVA was conducted to test the hypothesis that there would be one or more mean differences between AOC experience *vs.* no AOC experience and mental health outcomes, *i.e.*, levels of depressive symptoms and anxiety, complicated grief, and decision regret. A statistically significant MANOVA main effect of moderate size was obtained, Pillai’s criterion = .06, F (4,253) = 3.75, p = .006, *η*²p = .06 (**Figure 3**). Follow-up univariate Games–Howell tests to examine mean difference comparisons across all mental health outcomes, using Tukey’s method for adjusting p-values, showed that there was a significant difference of moderate size in decision regret between bereaved AOC caregivers compared to non-AOC caregivers, Cohen’s d = 0.49, 95% CI [0.23, 0.76] (**Table 3**). Multiple imputation of missing data did not affect these results: Pillai’s criterion = .05, F(4,219) = 3.17, p = .015, *η*²p = .05, for pairwise exclusion of participants. As before, follow-up tests revealed a significant difference in decision regret between AOC caregivers compared to non-AOC caregivers, Mean Difference = 9.90, t = 3.214, p = .001, Cohen’s d = .44, 95% CI [.17,.71].

**Table 3:** Means, Standard Deviations, and Games Howell Post-hoc Tests for Mental Health Outcomes Dependent on the Experience of Aggressive Care.

Measure	AOC^a^			non-AOC^a^			*t*-statistic	*df*	*Mean difference*	*95% CI^b^ for mean difference*	Standard error (SE)	adjusted *p*-value using Tukey’s method
	n	M	SD	n	M	SD						
Decision regret	84	43.3	20.89	184	32.6	21.93	3.79	167.0	10.17	[5.14, 16.3]	2.00	<.001
Complicated grief	82	48.3	15.33	182	44.7	13.29	1.84	141.0	3.52	[−0.26, 7.39]	1.37	.068
Level of depressive symptoms	83	9.95	6.48	184	9.19	5.91	0.81	147.0	0.76	[−0.97, 2.32]	0.59	.420
Level of anxiety	83	7.64	5.27	184	7.26	5.26	0.49	156.0	0.38	[−0.97, 1.78]	0.49	.563

^a^AOC, Aggressiveness of Care; ^b^CI, Confidence Interval.

The direction of the findings did not vary when we included gender of the bereaved, relationship to the deceased (spouse/partner *vs* other), the patient’s age at time of death, and the time since death and study participation as covariates (only main effects) in the MANOVA. In particular, the main effect of AOC on mental health outcomes remained significant, Pillai’s criterion = .06, F(4, 246) = 3.63, p = .007, *η*²p = .06. Except for relationship to the deceased, Pillai’s criterion = .06, F(4, 246) = 3.82, p = .005, *η*²p = .06, no significant main effects emerged (all p’s >.076). Univariate analyses of covariance showed that, as before, participants differed in decision regret between AOC groups, F(1, 259) = 14.5, p <.001, with individuals having experienced AOC reporting higher decision regret than those not having experienced AOC. There were no differences with respect to depressive symptoms, anxiety symptoms, and complicated grief (p’s >.29). Of the covariates, only relationship to the deceased emerged as a significant predictor of decision regret, F(1, 259) = 4.26, p = .040, with bereaved spouses and partners reporting moderately lower regret (M = 35.18, SD = 21.59) than others (M = 47.65, SD = 29.69, Cohen’s d = .559). None of the covariates significantly predicted one of the other mental health outcomes (p’s >.067) (data not shown).

## Discussion

### Key Results

In this study, we found that, according to their bereaved caregivers, three in ten cancer patients experienced some form of AOC at the end of their lives. While one in five started a new chemotherapy regimen within the last 14 days of their lives, one in seven was treated on an ICU in the last month of their lives which, in bereaved caregivers, is a known risk factor for developing mental health disorders (33). Bereaved caregivers who had experienced AOC for the patient suffered from higher decision regret and higher complicated grief compared to non-AOC caregivers, although the latter finding did not reach statistical significance.

### Limitations and Strengths

When interpreting the results, one must consider the following study limitations and strengths. First, due to the nature of the cross-sectional study design, we assessed AOC and mental health outcomes in bereaved caregivers only at one given timepoint and gave no indication of the temporal sequence (34). Hence, another timeframe may have provided differing results, and it is not possible to infer causality. However, we describe (a) the frequency of AOC and (b) its role as potential risk factor for decreased psychological functioning in bereaved caregivers. Second, our sampling frame heavily relied on the condition that caregivers had provided some form of contact detail so that we were able to address them. In the German health care system, there is no legal requirement that patients must indicate a contact person and provide and/or update contact details or even that attending staff has to inquire about these details. Therefore, often caregivers are not inserted in the patient’s medical record. Third, more than half of the eligible caregivers did not complete the questionnaires. Generally, non-response bias undermining the generalizability of the findings is a ubiquitous challenge, particularly in the hard-to-reach population of bereaved caregivers dealing with prolonged grief (35–37). Nevertheless, our response rate of 46.1% is in line or somewhat higher than those from other studies conducted in similar populations reporting response rates ranging from 13 to 62.4% (20, 38–40). However, the study with a response rate of 62.4% was conducted in the unique environment of US Veteran Heath Affairs (20). Furthermore, in that study caregivers who could not have been reached were defined as not-eligible. In contrast, in our study those for whom contact details were available but could not be reached were defined as potentially eligible. This led to a conservatively determined response rate. Notwithstanding, we employed all common techniques for minimizing non-response, *i.e.* telephone prompting, second and third mailing of questionnaires, and letters/calls highlighting the importance of replying. Although we were only able to include gender in the non-responder analysis, we did not find any statistically significant differences between non-responders and responders. Third, due to the nature of our sampling, we could not retrieve any information on non‐responding participants which impeded sensitivity analysis between participants and non‐respondents for the assessment of potential selection bias. However, since scores in our sample were sufficiently dispersed, we would rate the risk of selection bias relatively low. Additionally, the study was conducted at a high‐volume academic comprehensive cancer center and may therefore have overestimated the frequency of AOC to some extent. Finally, findings from studies with follow-back designs may be susceptible to recall bias (41). Caregivers may not have remembered the cancer treatment trajectory at end-of-life correctly (42). Nevertheless, there is ample evidence that proxies do reliably report observable service provision at the end of life (43). Since regulations on data protection, especially of medical data, are relatively strict in the German health care system, there is no nationwide medical record where we could have extracted this information. At any rate, we have prepared the ground for future cohort studies, which are required for evaluating our hypothesis longitudinally and with respect to long-term effects.

### Comparison With Prior Work

To the best of our knowledge, this is one of the first studies providing data on the frequency and impact on caregivers of AOC in Europe. The findings of our study are essentially in line with prior findings on AOC in cancer patients. The estimated frequency of AOC in our sample concurs with the frequencies of the different components of AOC reported in a recently published systematic review of 42 studies in patients with lung cancer (7). Although some of the criteria in the review were more liberal than ours (*e.g.*, any form instead of “new beginning” chemotherapy within the last 30 days, any admission to ICU instead of “more than one day” within the last 30 days), the reported ranges of frequencies of chemotherapy within the last 14 days and ICU admissions (ranges: 1–23.8% and 2–30%, respectively) entail the point estimates we found. However, compared with numbers from other studies reporting numbers for Europe and the US, our findings are mostly higher. Generally, a consistent difference in AOC frequencies cannot be observed between Europe and the US. One study identified similar frequencies of chemotherapy use and ICU admissions in cancer patients in Europe compared to the US (44). Furthermore, within both regions, AOC frequencies are not consistent, either (20, 45–47). Authors of those studies acknowledged these inconsistent results and raised methodological differences as sampling strategies (*e.g.*, different ages of patients) on the one hand and systematic differences as insurance structures and specific health care environments (*i.e.*, US veterans’ health care) on the other hand as possible explanations.

In our study, bereaved caregivers who had experienced AOC for the patient suffered from higher decision regret compared to non-AOC caregivers. This finding is in line with reports from a large sample of bereaved family members of Medicare patients with advanced-stage lung or colorectal cancer who, faced with the experience of AOC, were more likely to develop not only regret but also major depression (24). AOC is often related to a specific place of death (i.e. dying in a hospital or even an ICU), which is an important determinant of quality of death and dying as perceived by caregivers with in-hospital death entailing increased psychological distress for caregivers (22). Specifically, bereaved caregivers of patients who die on an ICU are more likely to develop a posttraumatic stress disorder compared to caregivers of patients who die at home or in a hospice (33). As there is still a lack of data on the impact of AOC on caregivers, especially in Europe, our results may contribute to the discussion on adverse or unintended effects of cancer treatment not only for patients but also for their caregivers, although our data are from 2016. While interventional approaches to reduce psychological distress in bereaved caregivers resulting from aggressive care have rarely been put forward, interventions targeting patients have been evaluated. Essentially, these interventions focus on the promotion of shared decision-making at end-of-life with physicians taking a proactive role. Specifically, two recent systematic reviews show that proactively initiated, structured communication (*e.g.* shared-decision making featuring discussions about treatment near to death between patients and physicians) may reduce the frequency of AOC and decision regret in patients (48, 49). A recent study suggests that, for caregivers, comparable effects are achieved by involving them regularly in discussions about treatment at end-of-life of cancer patients (50). According to these findings, making well informed treatment decisions may reduce the occurrence of mental distress in both patients and caregivers. As these studies included mostly non-European data, primarily from the US, there is a need to develop and evaluate these types of interventions for European health care systems.

## Conclusion

In this study, we found that, according to their bereaved caregivers, three in ten cancer patients experienced some form of AOC at the end of their lives. Bereaved caregivers who had experienced AOC for the patient suffered from higher regret regarding treatment decisions they were involved in compared to non-AOC caregivers. For clinicians aiming at best available care for both patients and caregivers, our findings call for a cautious course of action when it comes to potentially aggressive treatment decisions. Not only should the potential benefit always be weighed against potential adverse events/effects, but clinicians are encouraged to present and discuss treatment scenarios with both patients and caregivers whenever possible. Consequently, patients and caregivers may be able to make well informed decisions regarding the course of treatment.

Concerning future research on AOC, there is a need for longitudinal studies which may substantiate our findings but can also explore specific trajectories related to AOC as well as potential long-term effects. Cohort studies will (a) allow for the comparison of patients for whom chemotherapy was stopped earlier compared to patients who received AOC at end-of-life and (b) further clarify how dyadic aspects play out in the development of distress both in patients and caregivers. Especially in Europe, the linkage between AOC and its effect on caregivers’ mental health needs to be further investigated. In perspective, interventions targeting both patients and caregivers should be tailored, piloted, and evaluated. Finally, considering broader efforts to enhance care, health policymakers should pay closer attention to a regulatory framework (*e.g.*, reimbursement schemes, legislative concerning advanced care planning) that supports the involvement of caregivers early in the treatment trajectory.

## Data Availability Statement

The raw data supporting the conclusions of this article will be made available by the authors, without undue reservation.

## Ethics Statement

The studies involving human participants were reviewed and approved by the Institutional Review Board of the Medical Faculty of Heidelberg University (Registration-No. S-500/2014). The patients/participants provided their written informed consent to participate in this study.

## Author Contributions

MHar, DJ, H-CF, and MHau conceptualized the study. CB, NB, JT, and MHau collected, prepared, analyzed and visualized the data. JT, MHar, DJ, CB, H-CF, and MHau drafted the manuscript. All authors revised the manuscript critically for important intellectual content. All authors contributed to the article and approved the submitted version.

## Funding

Financial support for this study was provided entirely by a grant from the German Research Foundation (Deutsche Forschungsgemeinschaft, DFG) (Grant no. HA 7536/1-1). The funding agreement ensured the authors’ independence in designing the study, interpreting the data, writing, and publishing the report.

## Conflict of Interest

The authors declare that the research was conducted in the absence of any commercial or financial relationships that could be construed as a potential conflict of interest.

## Abbreviations

AOC, Aggressiveness of Care; DRS-C, Decision Regret Scale for Caregivers; GAD-7, Generalized Anxiety Disorder Scale; ICG, Inventory of Complicated Grief; ICU, Intensive Care Unit; MANOVA, Multivariate Analysis of Variance; NCT, National Center for Tumor Diseases; PHQ-9, Patient Health Questionnaire 9.
