# Supplementary material for: Aggressiveness of Care at the End-of-Life in Cancer Patients and Its Association With Psychosocial Functioning in Bereaved Caregivers
Source: Front Oncol. 2021 Jun 4;11:673147. doi: 10.3389/fonc.2021.673147 (PMC8212704; doi:10.3389/fonc.2021.673147)
Supplement: Supplementary file 1 [file DataSheet_1.docx]

**Supplementary material**

In the following we present the results for assumption checking prior to conducting the multivariate analysis of variance (MANOVA).

1. **Linearity and Additivity among the Dependent Variables**

*Experience with AOC group*


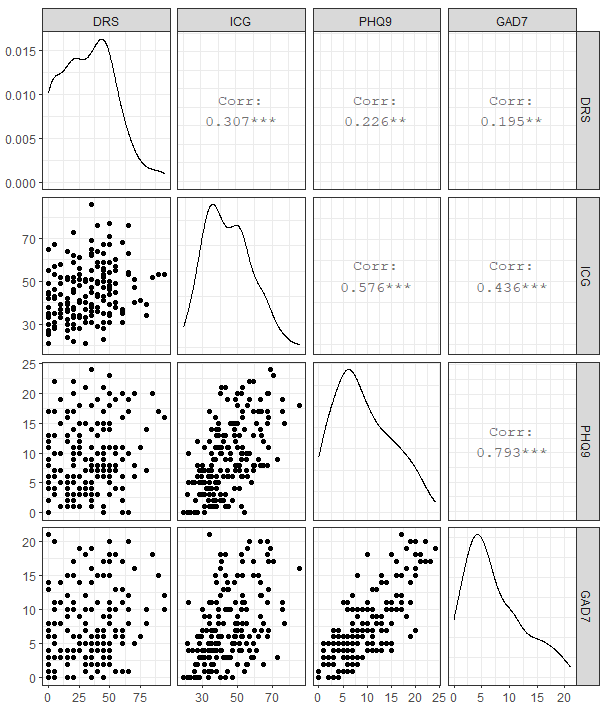


*No experience with AOC group*


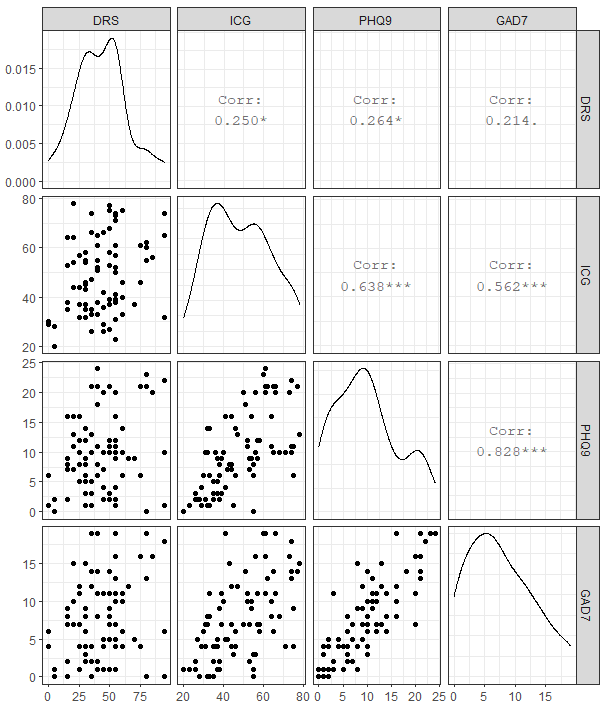


Inspection of scatterplot matrices by treatment groups pointed to linearity. Bivariate correlations were well below *r* = .90 ruling out multicollinearity. Bartlett’s test for sphericity indicated a significant degree of intercorrelation of the dependent measures, *χ²*(6, *N* = 292) = 458.81, *p* < .0001.

**B. Multivariate Normal Distribution for Dependent Variables**

*Decision Regret Scale (DRS-C)*


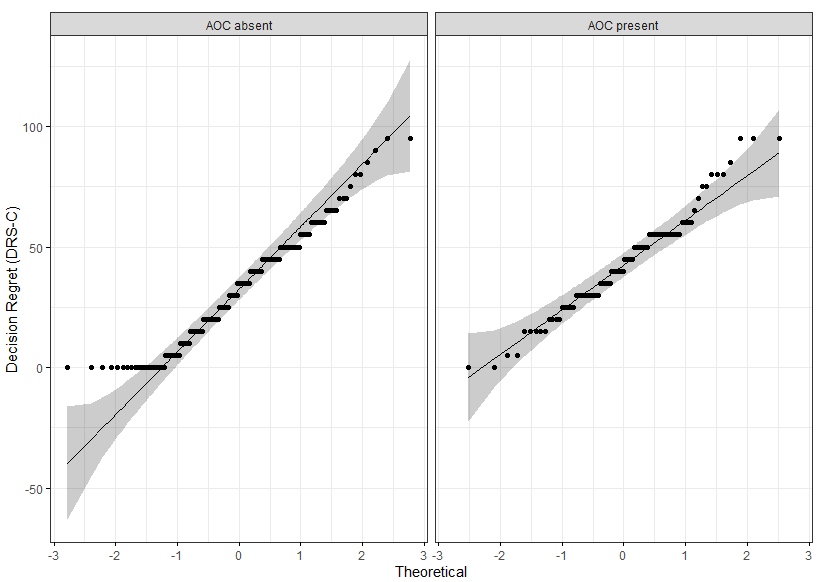


*Inventory of Complicated Grief (ICG)*


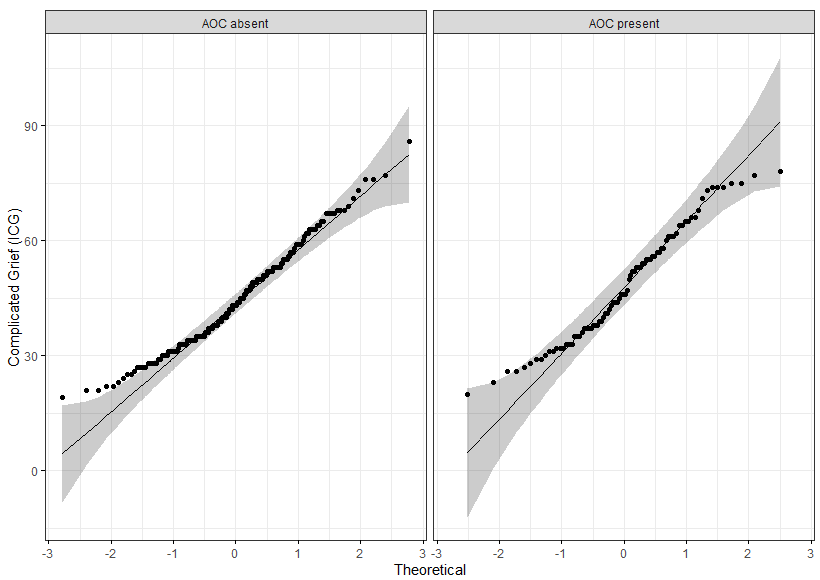


*Patient Health Questionnaire (PHQ-9)*


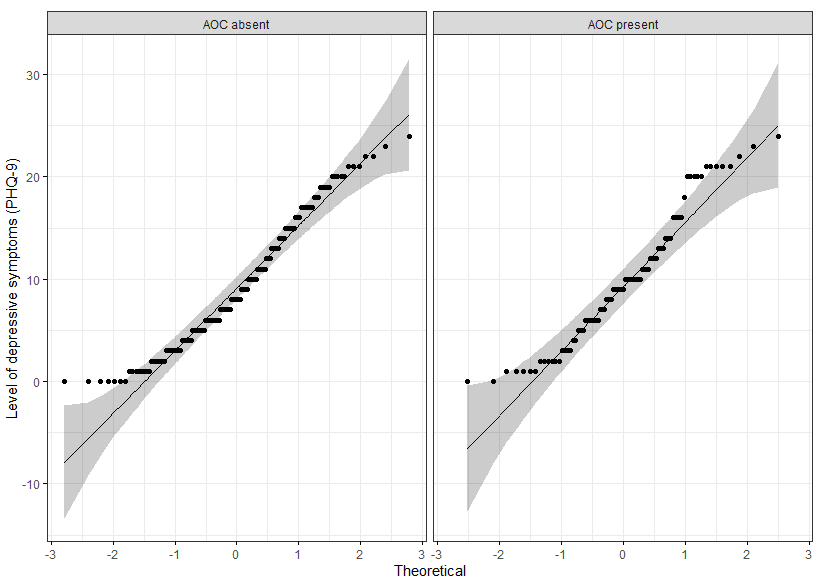


*Generalized Anxiety Disorder (GAD-7)*


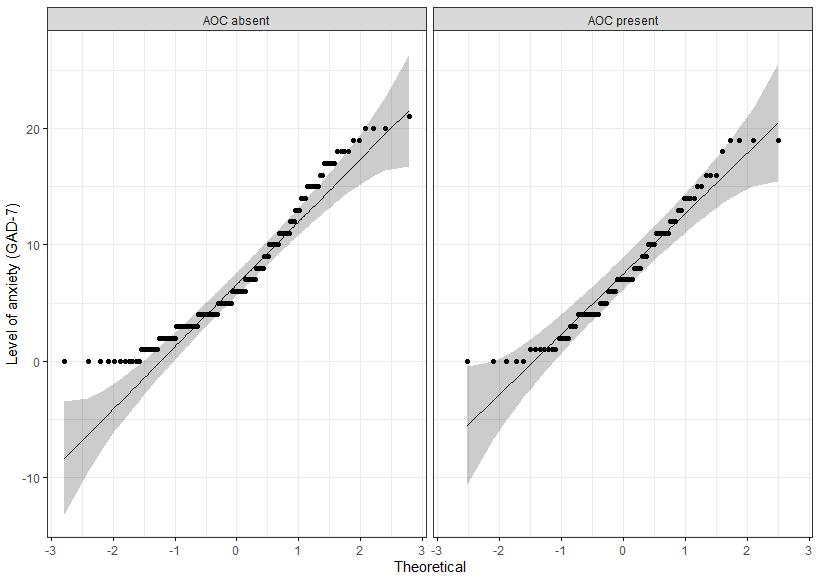


In addition to the graphical evaluation of the distribution of all numerical variables, we assessed multivariate normality based on Shapiro-Wilk W statistic. Data for the experience with AOC group (*n* = 89) were found to be approximately normally distributed (*W* = 0.98, *p* = .29), while data for the no experience with AOC group (*n* = 203) were not found to be normally distributed (*W* = .98, *p* = .023). However, we proceeded with our analysis since all dependent variables met the univariate normality requirement (skewness and kurtosis were below 2 and 7, respectively in each of the two groups) and, in this situation, departures from multivariate normality are usually inconsequential in terms of their impact multivariate test procedures used with MANOVA in larger sample sizes.^[[1]](#footnote-1)^

**C.** Equality of Variance–Covariance Matrices for all Treatment Groups

The assumption of homoscedasticity for each individual variable separately was met, given that all p > .001 in the Levene’s tests. The Box’s M-test for equality of covariance matrices yielded a nonsignificant result, *χ²*(10, *N* = 262) = 6.23, *p* = .80. The determinant of the covariance matrix was positive (102792.2). Hence, the assumption of homogeneity of variance-covariance matrices for the three variables collectively was met.

**D. Lack of Independence among Observations**

The ICC (single raters absolute, type ICC1) amounted to -.10 which indicated only a small correlation between variables. Hence, independence assumption was upheld. The Durbin-Watson (DW) test, DW =2.04, *p* = .75, was nonsignificant indicating that residuals from the MANOVA were independent, i.e., not autocorrelated.

1. Hair, J. F. (2019). Multivariate data analysis (Eighth edition). Cengage. [↑](#footnote-ref-1)
